# Mesenchymal stem cells: amazing remedies for bone and cartilage defects

**DOI:** 10.1186/s13287-020-02001-1

**Published:** 2020-11-23

**Authors:** Parisa Kangari, Tahereh Talaei-Khozani, Iman Razeghian-Jahromi, Mahboobeh Razmkhah

**Affiliations:** 1 Department of Tissue Engineering and Applied Cell Sciences, School of Advanced Technologies in Medicine, Shiraz University of Medical Sciences, Shiraz, Iran; 2 Tissue Engineering Laboratory, Department of Anatomy, School of Medicine, Shiraz University of Medical Sciences, Shiraz, Iran; 3 Cardiovascular Research Center, Shiraz University of Medical Sciences, Shiraz, Iran; 4 Shiraz Institute for Cancer Research, School of Medicine, Shiraz University of Medical Sciences, Shiraz, Iran

**Keywords:** Mesenchymal stem cells, Bone, Cartilage, Regeneration

## Abstract

Skeletal disorders are among the leading debilitating factors affecting millions of people worldwide. The use of stem cells for tissue repair has raised many promises in various medical fields, including skeletal disorders. Mesenchymal stem cells (MSCs) are multipotent stromal cells with mesodermal and neural crest origin. These cells are one of the most attractive candidates in regenerative medicine, and their use could be helpful in repairing and regeneration of skeletal disorders through several mechanisms including homing, angiogenesis, differentiation, and response to inflammatory condition. The most widely studied sources of MSCs are bone marrow (BM), adipose tissue, muscle, umbilical cord (UC), umbilical cord blood (UCB), placenta (PL), Wharton’s jelly (WJ), and amniotic fluid. These cells are capable of differentiating into osteoblasts, chondrocytes, adipocytes, and myocytes in vitro. MSCs obtained from various sources have diverse capabilities of secreting many different cytokines, growth factors, and chemokines. It is believed that the salutary effects of MSCs from different sources are not alike in terms of repairing or reformation of injured skeletal tissues. Accordingly, differential identification of MSCs’ secretome enables us to make optimal choices in skeletal disorders considering various sources. This review discusses and compares the therapeutic abilities of MSCs from different sources for bone and cartilage diseases.

## Introduction

Diseases of skeletal system are extensively widespread in aged population and are considered to be one of the main causes of disability and morbidity [1]. The most common disorders of the skeletal system include intervertebral discs (IVDs), osteoporosis, bone fractures, osteogenesis imperfecta (OI), osteoarthritis (OA), and rheumatoid arthritis (RA) [2] (Table 1). Among various therapeutic approaches for the treatment of these diseases, stem cell therapy seems to be more promising. Stem cells are introduced into tissues to repair, replace, and treat a defect with or without the addition of external gene. The origin of the stem cells can be from autologous or allogeneic sources. They can be used either as naive or primed of the desired lineage [17].

**Table 1:** List of the main skeletal diseases, their clinical description and molecular features

Bone diseases	Clinical description	Molecular features
**Intervertebral disc (IVD) degeneration**	Increased extracellular matrix breakdown and abnormal matrix synthesis leading to reduced hydration, loss of disc height, and decreased ability to absorb load, disc herniation, vertebral instability and spinal stenosis, back and neck pain [3]	Collagen I (COL1A1/A2), Collagen IX (COL9A1/A2/A3), CollagenXI (COL11A1/A2*/*A3),VDR genes (TaqI, ApaI), Col I (COLIA1, Aggrecan (CS1), MMP-3(5A/6A) [4, 5]
**Osteoporosis**	Acute back pain caused by a pathologic vertebral compression fracture as the earliest symptom, decreased density (mass/volume) of normally mineralized bone, decreased mechanical strength, making the skeleton more likely to fracture [6]	Col I (COL1A1/A2), PTH, PTHR, VDR, BMPs (BMP2,4,7, OP1LRP5), LRP6, RANK, RANKL [7]
**Osteogenesis imperfecta (OI)**	Progressive skeletal deformation, loss of BMD, frequent fractures, short stature, joint hypermobility and pain [8, 9]	mutations in the type I collagen genes COL1A1/A2, collagen modification (CRTAP, LEPRE1, PPIB), processing (BMP1), or folding (SERPINH1, FKBP10 [8, 10]
**Osteoarthritis (OA)**	Joint inflammation, joint pain, stiffness, swelling and restriction of joint function [11]	COL2A1, COL9A3, COL11A1, CRTM, VDR, ESR1, BMP5, ALDH1A2, MCF2L, CHADL, GDF5 and FILIP1, GLIS3, TGFB1, TNC and WWP2 [12–14]
**Rheumatoid arthritis (RA)**	Joint degeneration, loss of cartilage, and alterations of subchondral bone, abnormalities of weight-bearing joints and hands, including knees and hips, symptoms of OA including pain, stiffness, and altered function in knee and hips [15]	HLA-DR, PTPN22, IL6R, TRAF1/C5, STAT4, PADI4, FCGR, CD40, CCL21, CCR6 [16]

*COL* collagen, *VDR* vitamin D receptor, *MMP* matrix metalloproteinase, *PTH* parathyroid hormone, *PTHR* parathyroid hormone receptor, *BMP* bone morphogenetic protein, *LPR* low-density lipoprotein receptor-related protein, *RANK* receptor activator of nuclear factor kappa B, *RANKL* RANK ligand, *BMD* bone mineral density, *CRTM* cartilage matrix protein, *ESR* estrogen receptor, *CRTAP* cartilage-associated protein, *LEPRE1* leucine proline-enriched proteoglycan1, *PPIB* peptidyl-prolyl isomerase 1 (cyclophylin B), *SERPINH1* serpin peptidase inhibitor, clade H, *FKBP10* Fk506-binding protein 10, *ALDH* aldehyde dehydrogenase, *MCFL2* MCF.2 cell line derived transforming sequence-like protein, *CHADL* chondroadherin like, *GDF5* growth differentiation factor 5, *FILIP1* filamin-A-interacting protein 1, *GLIS3* GLI-similar 3, *TGFB1* transforming growth factor beta 1, *TNC* tenascin C, *WWP2* WW domain containing E3 ubiquitin protein ligase 2, *HLA-DR* human leukocyte antigen – DR isotype, *PTPN22* protein tyrosine phosphatase, non-receptor type 22, *IL6R* interleukin-6 receptor, *TRAF1/C5* tumor necrosis factor receptor-associated factor-1, *STAT4* signal transducer and activator of transcription 4, *PADI4* peptidylarginine deiminase 4, *FCGR* Fc gamma receptor, *CCL21* CC chemokine ligand 21, *CCR6* CC chemokine receptor 6

**Fig. 1:**
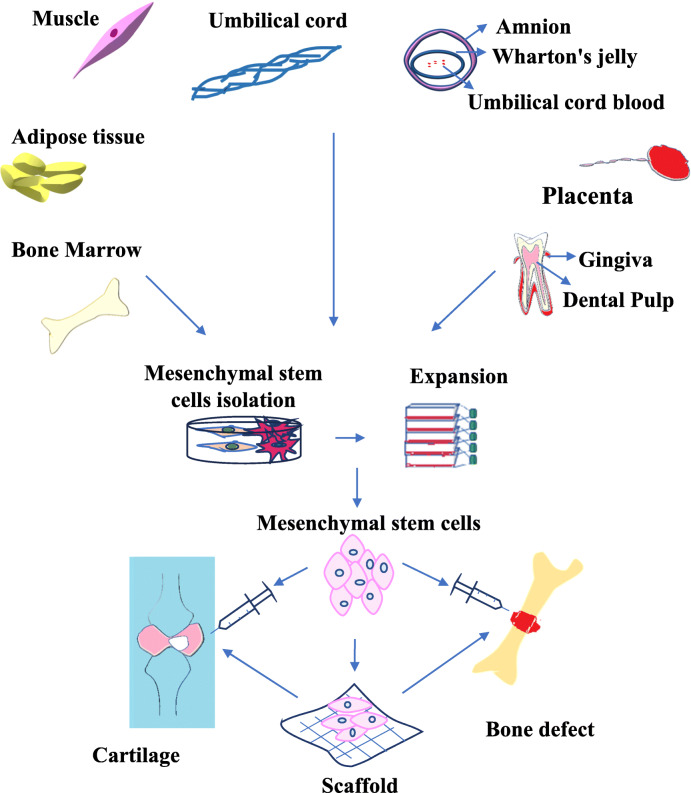
Mesenchymal stem cell (MSC) sources and applications. MSCs are originated from various sources such as bone marrow, adipose tissue, placenta, umbilical cord, Wharton’s jelly, muscle, and dental tissues. They may be used either by loading within scaffold or as cell suspensions for regenerative purposes including cartilage and bone defects

Stem cells are undifferentiated biological entities with the capacity to self-renew and differentiate into specialized cell types. Based on differentiation potential, they are classified as totipotent, pluripotent, multipotent, oligopotent, and finally, unipotent cells [18]. Mesenchymal stem cells (MSCs) are multipotent stromal cells with mesodermal and neural crest origin [19, 20]. They are the most commonly used stem cells in the current preclinical and clinical studies on skeletal diseases [21] (Table 2) either through direct injection or along with scaffolds (a range of natural gels and hydrogels based on collagen, hyaluronic acid (HA), glycosaminoglycans (GAGs), agarose, gelatin and alginate) [37–39] (Fig. 1). These cells are isolated from a variety of tissues like bone marrow (BM), adipose tissue, fetal liver, umbilical cord (UC), muscle, endometrial polyps, dental tissue, synovial fluid, skin, foreskin, Wharton’s jelly (WJ), placenta, dental pulp (DP), breast milk, gingiva, amnion, and menstrual blood [40–54]. MSCs are characterized as plastic adherent cells with fibroblastic morphology in culture. These cells lack the expression of hematopoietic markers such as CD45, CD34, and CD14, but express mesenchymal specific markers including CD73, CD90, and CD105 [55]. A list of positive and negative markers on MSCs from various sources is presented in Table 3. Human MSCs (hMSCs) usually express low levels of MHC class I, with no expression of MHC class II [64]. These cells demonstrate three distinct biological characteristics (potential of differentiation, secretion of trophic factors and immunoregulatory properties) which make them an excellent candidate for cell therapy [65]. MSCs possess the capacity to differentiate into a wide variety of cell types including adipocytes, osteoblasts, chondrocytes, and myocytes. Also, they are capable of trans-differentiating into ectodermal lineages such as neuronal cells and endodermal lineages such as hepatocytes and pancreatic islet cells [39, 65, 66]. MSCs are of great importance because of their paracrine effects through secreting growth factors and cytokines, such as vascular endothelial growth factor (VEGF), transforming growth factor beta (TGF-β), and interleukins (IL-1β, IL-6, and IL-8) [67]. Moreover, MSCs have an additional ability to modulate immune responses through repressing T cell proliferation, dendritic cell (DC) maturation, B cell activation, and cytotoxic activation of resting NK cells [68–73].

**Table 2:** Preclinical and clinical studies of MSCs for the treatment of skeletal diseases

Defect type	Model	MSC type	Findings
IVD	Porcine	Autologous BM-MSCs	Reduction in COL1 expression as a marker for fibrosis, reduction of inflammation marker IL1β and elevation of trophic factor BMP2, reducing disc degeneration [22]
	Rat	Xenogeneic hAD-MSCs	Viability and proliferative potentiate of AD-MSC transplanted within the rat IVD, contribution in the maintenance of disc height after the operation [23]
	Human (*n* = 5)	Autologous BM-MSCs	Improvement in strength and mobility post stem cell treatment [24]
	Human (*n* = 10)	Autologous BM-MSCs	Feasible and safe, rapid improvement of pain and disability (85% of maximum in 3 months) [25]
Osteoporosis	Goat	Autologous BM-MSCs	Improvement of bone formation in the osteoporotic model in vivo [26]
	Rat	Xenogeneic hUCB-MSCs	Enhancement of bone formation abilities in osteoporotic rat model similar to no osteoporotic bone regeneration [27]
OI	Mouse	Human fetal e-CSCs	Reduction of fractures, increasing bone ductility and BV by directly differentiating to osteoblasts, stimulating host chondrogenesis and osteogenesis [28]
	Human (*n* = 3)	Allogeneic BM-MSCs	Increase in total body bone mineral content and new dense bone formation [29]
Bone fractures	Rabbit	Autologous AD-MSCs	Improvement of healing process in tibial defects compared to using hydroxyapatite alone [30]
	Rat	Xenogeneic hDP-MSCs	Increased callus homogeneity, decline callus earlier size, increased percentage of lamellar in newly formed bone, lower incidence of fibrous tissue in the experimental group, advanced and more efficient bone healing in the cell-treated group compared to the control [31]
	Human (*n* = 18)	BMAC	Faster healing in BMAC cancellous bone allograft transplanted group compared to an autologous bone graft, efficacy of BMAC for treatment of nonunion [32]
OA	Rat	Allogeneic BM-MSCs	Chondroprotection and reduced subchondral bone mineral density in the transplantation [33]
	Human (*n* = 4)	Autologous BM-MSCs	Positive changes in all patients, clear bone formation in osteonecrosis patients, cartilage regeneration in the OA patients [34]
	Human (*n* = 6)	Autologous BM-MSCs	Improvement of pain, functional status of the knee and walking distance, increase in cartilage thickness, extension of the repair tissue and a considerable decrease in the size of edematous subchondral patches [35]
	Human (*n* = 18)	Autologous AD-MSCs	Reduced cartilage defects by regeneration of hyaline-like articular cartilage and improvement of function and pain of the knee joint without causing adverse events [36]

*IVD* intervertebral disc, *BM-MSCs* bone marrow-derived mesenchymal stem cells, *COL1* collagen typ1, *IL1β* interleukin1 β, *BMP2* bone morphogenetic protein, *hAD-MSCs* human adipose-derived mesenchymal stem cells, *hUCB-MSCs* human umbilical cord blood-derived mesenchymal stem cells, *OI* osteogenesis imperfecta, *e-CSCs* human fetal early chorionic stem cells, *BV* bone volume, *BMAC* bone marrow aspiration concentrate, *OA* osteoarthritis, *hDP-MCs* human dental pulp-derived mesenchymal stem cells

**Table 3:** Characterization of MSC from various tissues based on surface markers

Tissue	Positive markers	Negative markers
Bone marrow	CD29, CD31, CD44, CD49a, CD49b, CD49c, CD49d, CD49e, CD51, CD54, CD58, CD61, CD71, CD73, CD90, CD102, CD104, CD105, CD106, CD120a, CD120b, CD121a, CD124, CD146, CD166, CD221, CD271, SSEA-4, STRO-1 [56]	CD11a, CD11b, CD13, CD14, CD19,CD34, CD45, CD133 [56]
Adipose tissue	CD105, CD73, CD36, CD90, CD44, CD29, CD151, CD49d, CD44 [55, 57, 58]	CD45, CD34, CD14, CD11b, CD19, HLA-DR, CD34, CD38, CD31, CD106 [55, 57, 58]
Synovial fluid	CD9, CD10, CD13, CD44, CD54, CD55, CD90, CD105, CD166, D7-FIB, CD49a, CD147, CD73, PDGFRα (CD140a) [59]	CD14, CD45, CD34, CD117, CD62e, CD20, CD113, HLA-DR, CD68, CD31, ALP [59]
Dental pulp	CD29, CD44, CD105, CD146, CD117 and STRO-1 [60], SSEA-4, CD146, CD73, CD44, CD10, CD123 [61]	HLA-DR, CD106, CD34,CD7,CD31 [61]
Amnion	CD73, CD29, CD49f, Oct4, Nanog, Sox2, SSEA-3, SSEA-4, Rex1 [62]	CD14, CD20, CD34, CD45 [63]

### Bone structure

As the main part of the skeletal system, the bone contributes to the locomotion, soft tissue protection, harboring of BM, blood production, progenitor cell (mesenchymal and hematopoietic) housing, regulation of blood pH and maintenance of calcium, and phosphate homeostasis [74, 75]. Macroscopic examinations show that bone tissue is a heterogeneous and porous structure comprising two bone types including cortical (compact) and cancellous (spongy). Comparison of cortical and cancellous bones reveals significant different masses, so that the former has major mass-to-volume ratio [76, 77]. Owing to be a dynamic connective tissue, the bone has cells and extracellular matrix (ECM) which consists of organic and inorganic phases. Collagen fibers are the main makeup in the organic phase while inorganic phase is mainly composed of hydroxyapatite [76, 78, 79]. The cellular components are osteoprogenitors, osteoblasts, bone lining cells, osteocytes, and osteoclasts. Osteoblasts are one of the most important differentiated cells in the bone originating from bone marrow mesenchymal stem cells (BM-MSCs). Osteoblasts play critical roles in the synthesis of ECM components including type I collagen, proteoglycans, and non-collagenous proteins and also participate in matrix mineralization and blood-calcium homeostasis. Osteocytes, as the most abundant and long-lived cells, are the mature trapped osteoblasts in the lacunae. Osteoclasts are large multinucleated cells that originate from mononuclear cells and participate in the absorption of bone, calcium and phosphate excretion, bone healing, and remodeling [78, 80–83].

Bone diseases are one of the most common body injuries, and are associated with high health expenses exceeding billions of dollars annually [84]. Prevalence of such defects is increasing; thereby, they are considered an epidemic health challenge [85]. The bone benefits from the ability to repair itself throughout the life. Bone regeneration is a process in which osteoclasts and osteoblasts are tightly involved [80]. Despite spontaneous regeneration potential, there are several different reasons such as bone defect size and infection that cause damaged bone not to be able to restore itself [86]. In the following sections, common approaches and new therapies in restoring and treating bone defects will be discussed.

### Bone diseases and MSC therapy

#### Intervertebral disc (IVD) degeneration

Intervertebral discs (IVDs) are circular pieces of gelly fibrocartilage tissue between vertebral of the spine functioning for shock-absorption. They are the reason of flexibility in the neck and lumbar regions and thus contribute to motion. Anatomically, they have three important substructures: nucleus pulposus (NP), anulus fibrosus (AF), and cartilaginous end plates (CEPs) [87–89]. One of the highest risk factors for disc degeneration is aging [90]. As age increases, cellular and structural changes in NP, AF, and CEP lead to IVD [91]. Findings showed that IVD alterations during aging start cleft formation in NP [92]. Meanwhile, AF becomes disorganized, stiffer, and weaker [93].

One of the main symptoms of disk degeneration is back and neck pain which can terminate into several disabilities [93]. Treatment approaches for degenerative disc disease (DDD) are physiotherapy, pharmacotherapy, and surgery. However, they are only pain-relieving strategies in most cases and do not eliminate the underlying reason or restore the lost functions. Therefore, researchers are looking for novel therapeutics in order to regenerate DDD [88, 94, 95]. The best defining characteristics of DDD are the accumulation of senescent cells as well as reduction in the number of functional cells [95]. Several in vitro and in vivo studies on the degeneration of the IVD both in animal models and in clinical trials indicated that NP, AF, and CEPs contain cells with surface markers, morphology, proliferation rate, and multilineage differentiation capability similar to stem cells. This evidence suggests that IVDs possess stem cells that may provide cell candidates for cell-based regenerative medicine and tissue engineering [96–100]. On the other hand, studies on human degenerative IVD tissue demonstrated the existence of progenitor cells similar to BM-MSCs. As an endogenous source, they can be activated in situ after exposure to some growth factors involved in the repair of degenerative IVD [101, 102]. Regeneration is aimed to replace damaged with new functional cells that can be supplied by resident stem cells proliferation or division of differentiated cells. Therefore, these cells should be infiltrated into the tissue following by exposure to the growth factors until enabling to reconstruct lost structures [103–105]. Despite avascular nature of the IVD, it has been demonstrated that stem cells are capable of migrating from their niche throughout the body toward all three IVD constituent layers and they have more tendency to home at the vascular tissues such as CEPs and outer layer of AF [106–108]. Due to the absence of the active cell population in IVD, it needs to introduce stem cells in IVD for tissue regeneration. Findings from several preclinical and clinical studies demonstrated that MSCs are attractive candidates for regeneration of diseased disk [25, 109–111]. These cells should be isolated from an appropriate tissue and expanded in vitro and then, either intact or manipulated, implanted in the injured site [37, 112–114]. Multiple factors should be considered for choosing the appropriate cell source such as abundance, ease of obtaining, the capacity to differentiate into NP and AF cells, cell viability under hypoxic condition, cell viability under hypoglycemic condition, and non-tumorigenicity [109]. The investigations showed that scaffolds are capable of inducing MSC differentiation into a chondrogenic lineage such as NP-like cells under hypoxic or physiological conditions [115–117]. In an ex vivo study on degenerative IVD of bovine origin, it is demonstrated that human BM-MSCs (hBM-MSC) have immunomodulatory and anti-inflammatory effects through reduction of pro-inflammatory cytokines such as IL-6, IL-8, and tumor necrosis factor alpha (TNF-α) [118]. Transplantation of autologous BM-MSCs in a porcine model led to the elevation of trophic factor, bone morphogenetic protein-2 (BMP-2) in the NP, whereas the inflammation marker, IL-1β, was reduced in the AF [22]. On the other hand, Sun and co-workers, in an in vitro study first showed the impact of adipose-derived mesenchymal stem cells (AD-MSCs) for protecting human NP cells, through inhibiting caspase-9 and caspase-3 activity. Also, they revealed the suppression of pro-inflammatory factors, thereby preventing apoptosis and degeneration of NP cells. It was concluded that AD-MSCs may be a promising treatment strategy for DDD [119]. Recently, numerous clinical trials on regeneration of disk disease by MSC therapy are ongoing [25, 120, 121]. Yoshikawa et al. explored the role of autologous BM-MSCs in 2 patients with low back pain, leg pain, and numbness. They observed improvement of pain and disability during 3 months beside augmentation of hydration within one year after MSCs injection [121]. In addition to BM-MSCs and AD-MSCs [122–124], there are other major cell sources used for DDD regeneration including muscle-derived stem cells (MdSCs) [125], olfactory membrane stem cells [126], and synovial stem cells [127]. However, AD-MSCs and BM-MSCs are common sources for IVD regenerative therapy and BM-MSCs are widely applicable in human trials.

#### Osteoporosis

Osteoporosis, as a systematic skeletal disorder, is a common age-related bone defect which affects women more than men. Osteoporosis cause bone mineral density (BMD) loss and the degradation of the bone microstructure due to an abnormal imbalance between bone formation by osteoblasts and bone resorption by osteoclasts [128, 129]. Additionally, MSCs population in the BM are declined with aging; thus, their function will be limited and they cannot contribute to bone formation any longer [130]. Osteoporosis is of great importance mostly because of its effect on bone fragility. It also causes back pain and decreased quality of life which are collectively associated to high economic burden. Accordingly, suitable treatment strategies are essential for preventing disease and improving quality of life [131, 132]. Current osteoporosis treatments are principally using drug-based agents that usually stimulate apoptosis in osteoclasts and prevent the bone resorption [133]. However, they are associated with some side effects and therefore do not provide patient satisfaction [134–138].

During the last decade, stem cell therapy, as a new technology, is widely developed for bone regeneration in patients with osteoporosis. MSCs are the most extensively used stem cell type for this disease [128]. Studies in animal models have revealed that both allogeneic and autologous BM-MSCs transplantation are applicable for the treatment of osteoporosis [26, 139–142]. Allogeneic BM-MSCs therapy in glucocorticoid-induced osteoporosis in mice models showed osteoblastogenesis and promoted bone formation [143]. In a clinical trial by Lozano-Rivas and co-workers on new osteoporotic fractures, reduced pain was seen in patients with osteoporosis following autologous intravenous (IV) infusion of fucosylated BM-MSCs [144]. MSCs from perinatal tissues like human umbilical cord (hUC), human umbilical cord blood (hUCB), amnion, and chorion, have attracted special attention for osteoporosis improvement and preventing bone loss [145–147]. Recently, ovariectomy-induced osteoporosis was established in the rats along with reduction in estradiol level, bone mass, and collagen content. These rats received definitive number of human umbilical cord-derived mesenchymal stem cells (hUC-MSCs) and showed higher bone mass, collagen content, and osteoblasts number, while the number of osteoclasts decreased in the hUC-MSCs implantation site. Also, an in vitro study confirmed that hUC-MSCs promote osteoblasts formation while preventing the cellular activity of osteoclasts. This research showed that transplanted hUC-MSCs in the injured site in ovariectomized rats are capable of differentiating to osteoblasts and elevating collagen and osteocalcin levels as the main bone markers [148]. Hendrijantini and co-workers observed increase in the number of osteoblasts and overexpression of both TGF-β1 and runt-related transcription factor 2 (Runx2) after injection of hUC-MSCs in osteoporotic rat models [149]. Increased expression of TGF-β1 contributes to MSCs mobilization to the defect site, osteoblast differentiation, and ultimately bone formation [150, 151]. The osteogenic transcription factor, Runx2, prevents MSC differentiation into the other lineages except osteoblasts and enhances osteocalcin expression as a bone formation marker [151, 152]. These findings provide a new therapeutic strategy and demonstrate that hUC-MSCs can clinically resolve bone-related medical conditions such as osteoporosis.

AD-MSCs are more abundant and easily available in comparison with BM-MSCs. Indeed, their number is not affected by age making them more applicable in cell based therapeutics and tissue repair like osteoporotic bone regeneration [153, 154]. Comparing systemic injection of osteoporotic donor-derived AD-MSCs and BM-MSCs to ovariectomized mice indicated that AD-MSCs retained their anti-inflammatory potential and caused the maintenance of bone homeostasis in recipients with osteoporosis. AD-MSCs but not BM-MSCs showed the ability to resist in damaged microenvironment and maintain many properties including stemness and regulation of T cell viability. These results may show the priority of AD-MSCs over BM-MSCs for osteoporotic cytotherapy [155]. Oommen and co-workers suggested that AD-MSCs are useful treatment options for osteoporosis since these cells caused osteogenic induction by osteoblast differentiation and osteoid formation in ovariectomized rats [156]. In another study, Saito and his colleagues observed that autologous transplantation of BM-MSCs derived from osteoporotic rat models was associated with decreased osteoclast proliferation and mobilization, while adding UC extract improved the functionality of BM-MSCs regarding excessive osteolytic properties of osteoclasts [146]. Although BM-MSCs play key roles in maintaining bone metabolism, homeostasis, bone repair, and homing after systemic injection, their regenerative ability may be weak in the case of patients with postmenopausal osteoporosis [146, 157, 158]. Comparative study between different hMSCs sources including BM, AT, WJ, and placenta (PL) indicated that WJ-MSCs are the strongest inhibitors of T cell proliferation with less immunogenic effects compared with AD-MSCs, BM-MSCs, and PL-MSCs. Nevertheless, hWJ-MSCs had the lowest potential in osteogenesis than that of the PL-MSCs, AD-MSCs and BM-MSCs [159]. Due to the similar features to BM-MSCs including phenotypic characteristics, growth properties, differentiation capacities, secretory protein profiles, and low immunogenicity, perinatal derived MSCs are known as appropriate alternative sources for bone defect repair in patients with osteoporosis [160]. Overall, much more work seems to be needed to identify the appropriate stem cell source for clinical applications in osteoporosis.

#### Osteogenesis imperfecta (OI)

Osteogenesis imperfecta (OI) is a heterogeneous prenatal genetic disorder due to mutations in procollagen type I genes (COL1A1/A2) encoding the alpha1 and alpha2 chains of collagen type I which deteriorate the synthesis of this protein by osteoblasts. OI is characterized by progressive skeletal deformation, loss of BMD, frequent fractures, short stature, joint hypermobility, and pain [8, 9]. There is no definitive cure for OI at present, and current therapies are most effective in reducing disease severity. A favorable therapeutic method should replace dysfunctional cells with normal osteoblasts along with prohibition of osteoclast activity for the healthy bone formation [9]. Recently, the preclinical and clinical studies have indicated the successful intrauterine or multi-local engraftment of human fetal (HF) and adult MSCs in both mouse and human model of OI and successful differentiation of transplanted cells into functional osteoblasts [161–163]. Jones et al. suggested the PL as a practical source of stem cells for the treatment of OI based on their study showing that intraperitoneal (IP) injection of human fetal early chorionic stem cells (e-CSCs) in mice models caused the creation of osteoblasts and production of main proteins such as collagen as well as an increase in bone thickness and bone strength [28]. In a mouse model of OI, intrauterine transplantation of human fetal blood MSCs led to an enhancement of osteogenic genes expression such as osteocalcin, osteoprotegerins (OPG), osterix (OSX), and BMP2. The majority of donor cells have tended to migrate to the damaged area in bone and differentiate into collagen type Iα2 producing mature osteoblasts [164]. Le Blanc et al. used allogeneic male human fetal mesenchymal stem cells (hf-MSCs) to treat severe OI through intrauterine (IA) MSCs transplantation in female fetus in the 32nd week of gestation. Based on the results, engrafted hf-MSCs were able to differentiate into bone in a human fetus [163]. Transplantation of allogeneic BM-MSCs to children with OI demonstrated retention in one or more sites, including bone, skin, and marrow stroma, and acceleration of growth velocity during the first 6 months after transplantation [162]. Recent research showed that the clinical application of fetal MSCs is constrained due to their limited number and low availability. In contrast, e-CSCs are isolated in high numbers from the placenta during ongoing pregnancy without ethical restrictions [28]. Therefore, adult stem cells are safe for using in clinical trials without inherent limitations pertinent to embryonic stem cells.

### Bone fractures

A bone fracture or an osteotomy is one of the most common injuries among all people particularly elders and children. Every fracture in each site causes individual physical disability, low social efficiency, and imposing financial pressure [165, 166]. Bone fracture occurs under the circumstances of continuous mechanical stress, trauma, and some diseases such as osteoporosis and cancer [74]. Healing of this type of injury is a complex regenerative process with the involvement of numerous cell types including progenitor, inflammatory, endothelial, and hematopoietic cells as well as growth factors such as TGF-β [166, 167]. An effective treatment method for bone repair requires three biological properties: osteoinduction, osteoconduction, and osteointegration [168]. There are numerous therapeutic strategies such as natural bone grafts, using synthetic inorganic substitutes like calcium sulfate, calcium phosphate cements (CPCs), β-tri-calcium phosphate (β-TCP), and polymer-based bone substitutes (e.g., polylactic acid (PLA), poly(ε-caprolactone) (PCL)) for bone fracture repair. These methods are associated with some limitations like invasive surgical procedures, pain, and subsequent complications [169–172]. Researchers investigated new therapeutic approaches for overcoming these challenges and providing higher osteoconductivity. They suggested cell therapy as the best alternative for healing of fractured bone. In this regard, MSCs are one of the most available stem cell sources in bone repair [173, 174]. Generally, safety and efficacy of MSCs from different tissue sources including adipose tissue [30, 175], BM [176, 177], UCB [178], DP [31], and periosteum [179] for fracture regeneration were investigated in animal models. These studies reinforced the beneficial contribution of MSCs from different sources in the bone fracture repair either by differentiation into osteoblasts or through inhibition of inflammatory mediators. Acceleration of bone repair has initially been observed after IV injection of BM stem cells in a mouse model [180]. It is indicated that the controlled delivery of MSCs through biodegradable scaffolds can increase and accelerate the formation of functional new bone [181]. The scaffolds are 3D structures that promote cell adhesion, survival, migration and proliferation, accelerate bone remodeling, provide osteoconductive structural guidance, and in some cases act as the carrier [182]. Marcacci and coworkers were the first to report promising results using autologous in vitro expanded MSCs seeded onto a porous ceramic scaffold of hydroxyapatite (HA), which perfectly fitted the bone injured areas of four patients suffering from large bone diaphysis defects [183].

In addition, several clinical trials at different phases (I, II, or III) have been registered for bone fracture repair using BM-MSCs, AD-MSCs, hUC-MSCs, and human amniotic epithelial cells (ClinicalTrials.gov) which were implanted either via direct injection or after seeding them onto an osteogenic matrix. The required number of cells needed for fracture repair depends on the specific fracture characteristics, cell source, stimulation method, differentiation state, and using biomaterials. A comparison between three main sources of stem cells used to repair bone fractures suggested that isolation efficiency was higher from adipose tissue compared to other sources with respect to cell yield and feasibility. Although the ability for osteogenic differentiation seems to be higher in periosteum-derived mesenchymal stem cells (PD-MSCs), the most widely used cell source is yet allocated to BM for bone fracture repair strategies in recent clinical trials [166].

### Cartilage disorders and cell therapy

Cartilage as a strong supportive connective tissue is found in many areas of the human body including ribs, nose, ear, trachea, and IVD and is an important component of the joints [184–186]. It has dense and highly organized ECM embedding chondrocytes. Collagen type II is the main structural protein in cartilage and forms a meshwork for entrapping proteoglycans such as aggrecan, decorin, and sendycan [187, 188]. Aggrecan and other proteoglycans cause this framework to bound to the water and provide cartilage with tensile strength and flexible construct through which it can act as a supportive structure, maintain the shape, or absorb shock during physical exercise [189]. Three types of cartilages are hyaline (articular), elastic, and fibrocartilage that possess an avascular structure leading to a hypoxic environment with little capacity for self-repair, especially in the case of severe damage due to trauma or age-related degeneration [138, 142]. Hyaline cartilage is the most abundant type present on the articular surfaces of synovial joints providing a smooth, lubricated surface for articulation and facilitating the transmission of loads with a low frictional coefficient [136, 139]. Chondrocytes are spherical cells in a lacuna within matrix that produce and maintain cartilage architecture and remodel biochemical composition in response to changes in their chemical and mechanical environment in order to regulate cartilage homeostasis [140]. With age, chondrocytes naturally undergo senescent phenotypes and their responsiveness to growth factors reduces which results in accelerating cartilage disruption, cartilage matrix damage, and corresponding diseases [190]. Also, trauma, some diseases, and continual mechanical loading are other important factors for cartilage damage [142]. Due to the limited self-healing capacity of human cartilage, the repair of cartilage defects gives rise to a challenging clinical problem and cartilage regeneration has always been a key therapeutic target for treating articular cartilage damage in particular [139]. In the following sections, common approaches and new therapeutic strategies will be discussed in restoring and treating cartilage defects.

### Osteoarthritis (OA)

Osteoarthritis (OA) is one of the most common arthritis-related chronic disorders characterized by articular cartilage degeneration, thickening of subchondral bone, and osteophyte formation [191–193]. Disability of chondrocytes to produce sufficient functional matrix in order to repair damaged matrix is one of the prominent features of osteoarthritis [194]. OA can be established by activation of matrix proteases which affect joints in the knees and elbow, leading to joint pain, stiffness, swelling, and limitation of joint function [11, 189]. Studies have shown that aging, female gender, obesity, and osteoporosis are significant risk factors associated with OA [195].

Because of the limitation of self-healing capacity of articular cartilage, OA is one of the most challenging joint diseases. Most conventional treatments for OA such as physical therapy, drug therapy, and surgery are essential to manage the pain, stiffness, and swelling but are not effective to prevent the OA progression [196, 197]. Modern advances in regenerative medicine offer novel methods to treat OA. In recent years, cell therapy, especially with stem cells, is applied for the regeneration of OA damages [198]. By virtue of high proliferative capacity, chondrogenic differentiation capability, and immunosuppressive activities of stem cells, MSC-based therapies have demonstrated acceptable efficacy in cartilage repair in animal and clinical studies [199]. MSCs from various tissues such as adipose tissue [200], BM [201], synovial membrane [202], hUCB [203], and WJ [204] have been considered in different animal models. Overall, the results of investigations demonstrated that MSC-based therapy encourages pain reduction and OA improvement [194] mostly due to the differentiation capability of MSCs. It is demonstrated that both TGF-β1 and insulin-like growth factor 1 (IGF-1) act synergistically to stimulate MSCs’ chondrogenic differentiation [205].

BM and adipose tissue are common sources of multipotent cells for regenerating and repairing of an injured tissue. In order to evaluate the repair potential of BM-MSCs in OA, researchers showed that intra-articular (IA) injection of BM-MSCs in focal cartilage defects in immunocompetent transgenic rat can lead to collagen and matrix formation [206]. A phase I/II trial indicated that BM-MSCs injection in patients with knee osteoarthritis was associated with cartilage biomarker expression, reducing synovial inflammation, pain, and symptom mitigation, along with no serious adverse events [207]. Also, BM-MSCs exposed to bioactive factors loaded into a sponge composed of a hyaluronan derivative showed chondrogenic differentiation [208]. On the other hand, AD-MSC transplantation in the knee increased the synthesis of glycosaminoglycan, endogenous chondrogenesis supplemented by inflammation reduction, improvement in pain, function, and cartilage volume [209, 210]. MSCs from WJ and hUCB compared to AD-MSCs and BM-MSCs have many advantages such as higher proliferation rates, greater expansion ability, higher purity, abundant supply, and inexhaustibility for therapy [211–213]. ECM components in WJ are very similar to those of cartilage ECM. hWJ-MSCs express aggrecan, type II collagen, and SOX-9 as chondrocytes do [213]. Also, hWJ-MSCs express cell growth factors, chemokines, and cytokines at similar levels to those of cartilage. These results suggest hWJ-MSCs as appropriate cell candidates for OA’s cell therapy [214]. Moreover, the results of xeno-transplantation studies showed that human umbilical cord blood-derived mesenchymal stem cells (hUCB-MSCs) are less immunogenic and have higher chondrogenic differentiation potential, therefore promoting cartilage repair without bone formation for a long time [81, 84, 203, 215].

### Rheumatoid arthritis (RA)

Rheumatoid arthritis (RA) is one of the autoimmune inflammatory diseases of the joints presented with an imbalance of both the innate and adaptive immune systems. This disease leads to cartilage and bone degradations, causing pain and stiffness and may occur in other sites including tendon sheaths and bursae. The prevalence of this disease is approximately 0.5–1% in adults aged 40–50 years and is more common among women than men [216, 217]. Recently, MSC-based therapies have been suggested as favorable therapeutic approaches for inflammatory cartilage injuries such as RA. MSCs participate in cartilage regeneration after implantation into the injury site and differentiate into chondrocytes [218]. Moreover, MSC therapy reduces pathogenic T cell subsets such as Th1/Th17 cells in the collagen-induced arthritis (CIA) model [219, 220]. Studies showed that MSCs play an important role in inducing apoptosis of activated T cells via the Fas ligand (FasL)/Fas signaling pathway in arthritis disease [221]. Also, these cells promote immune modulation in RA by suppressing the expression of pro-inflammatory cytokines such as interferon gamma (IFN-γ), TNF-α, and matrix-degrading enzymes such as collagenase and gelatinase [222]. Evaluation of BM-MSC therapy on the healing of joints in animals with induced RA demonstrated that inflammation, joint swelling, and destruction of cartilage reduced significantly compared with an arthritic non-treated group [223]. Park and coworkers in the first human trial of hUCB-MSCs in patients with RA observed no major toxicity, serious adverse event, or major abnormalities in serum chemicals or hematologic profiles, both during and after the treatment [224]. Allogenic UC-MSCs transplantation in mice model of RA prevented arthritis progression by suppressing T follicular helper (Tfh) cells proliferation [220].

The investigations showed increased osteoclastic bone resorption as an important factor in the pathogenesis of RA [225]. An experimental study indicated that human gingival tissue-derived MSCs (G-MSCs) inhibit osteoclastogenesis in vitro and in vivo partially via CD39-CD73-adenosine signals and have therapeutic effects on bone erosion during CIA in vivo [226]. IV injection of hAD-MSCs in mice with RA reduced the level of pro-inflammatory cytokines while increased the level of anti-inflammatory cytokines with an induction in the number and function of regulatory T cells (Tregs) both in the peripheral blood and in the spleen [227]. Recently, a meta-analysis study compared the effects of MSCs derived from different tissue sources showing that hUC-MSCs, hAD-MSCs, and G-MSCs have better treatment effects on RA compared with stem cells from other origins, such as BM [228].

#### Regeneration mechanisms of mesenchymal stem cells in defected bone and cartilage

**Fig. 2:**
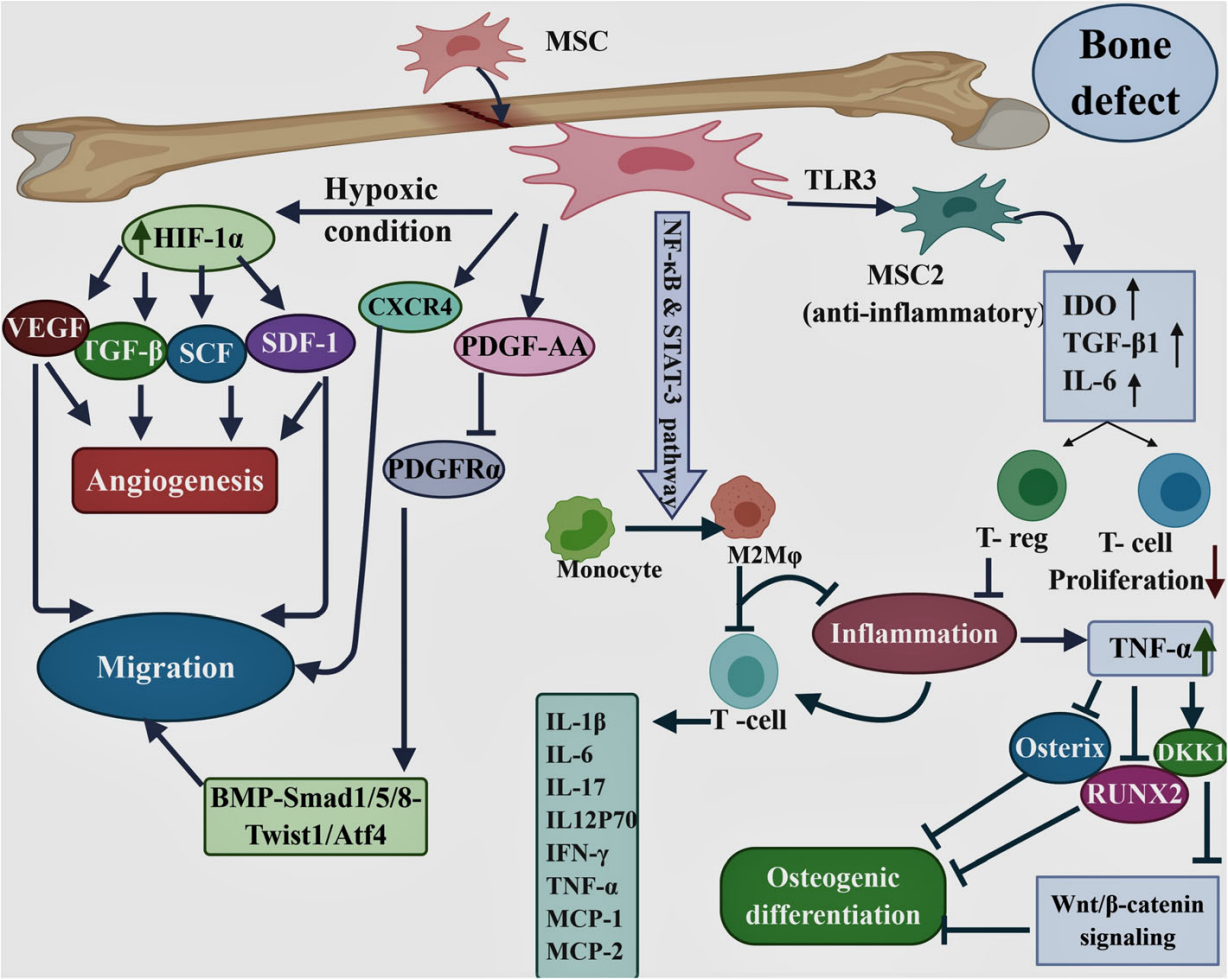
Schematic summarizing the mechanisms of repairing bone by MSCs. The figure was designed using the web-based tool BioRender. Mesenchymal stem cells (MSCs) contribute to bone regeneration by several mechanisms including migration, angiogenesis, response to inflammation condition, and differentiation through production of a variety of mediators. Hypoxia-inducible factor 1-α (HIF-1α), stem cell factor (SCF), transforming growth factor-beta (TGF-β), vascular endothelial growth factor(VEGF), stromal cell-derived factor (SDF)-1, and CXC chemokine receptor (CXCR) 4, platelet-derived growth factor (PDGF-AA), platelet-derived growth factor receptor-alpha (PDGFRα), Toll-like receptors (TLRs), nitric oxide (NO), indoleamine 2,3-dioxygenase (IDO), regulatory T cell (T reg), nuclear factor kappa-B (NF-κB), signal transducer and activator of transcription 3 (STAT-3), interferon gamma (IFN-γ), tumor necrosis factor alpha (TNF-α), monocyte chemoattractant proteins-1 (MCP-1), macrophage inflammatory protein-1(MIP-1), Dickkopf 1(DKK1), runt-related transcription factor 2 (RUNX2), M2 type of macrophage (M2MQ)

Several in vitro and in vivo studies indicated that MSCs, as the most commonly used stem cells in regenerative medicine, involve in the bone healing process because of their potential to increase osteoinduction and osteogenesis [229, 230]. These cells can play crucial roles in bone repair and regeneration by several mechanisms (Fig. 2) including facilitating cell migration, homing, angiogenesis, response to inflammation, and differentiation [231].

MSCs have the migration and homing ability into injured sites that are considered as the primary steps for bone formation and defect repair in MSC-based therapy. The recruitment of MSCs is initiated by the response of MSCs to inflammatory factors released from the bone fracture site. These processes are affected by intracellular signaling and interaction between chemokines, chemokine receptors, adhesion molecules, and proteases [232, 233]. Platelet-derived growth factors (PDGFs) and bone morphogenetic proteins (BMPs) play critical roles in bone development and bone fracture healing [234]. PDGF-AA is able to activate BMP-Smad1/5/8 signaling through downregulating platelet-derived growth factor receptor-alpha (PDGFRα) and promotes MSC migration via BMP-Smad1/5/8-Twist1/Atf4 [235]. Due to vascular damage and hypoxic condition in the injured site, expression of some growth factors such as hypoxia-inducible factor 1-α (HIF-1α) increases the production of the stromal cell-derived factor-1 (SDF-1) in the cells of damaged bone. Also, it mediates the expression of its receptor CXC chemokine receptor 4 (CXCR4) in MSCs [236–239]. Therefore, SDF-1/CXCR4 axis promotes the mobilization of MSCs to the defect site and enhances bone regeneration [240]. Expression of CXCR4 and Cbfa1 (core binding factor alpha 1, also called Runx2) increased MSC homing and promoted bone formation after four weeks of transplantation [158]. In vitro and in vivo studies showed that TNF-α, as one of the main proinflammatory cytokines, induces the expression of LRG1 through p38 and nuclear factor kappa-B (NF-κB) signaling to promote angiogenesis and MSC migration [241].

Transplanted MSCs can contribute to bone regeneration through angiogenesis stimulation [242]. hMSCs reside in hypoxic perivascular niches [243], express HIF-1α in response to hypoxic condition in defect site [244–247], and induce the expression of angiogenic factors such as VEGF, TGF-β, SDF-1, and stem cell factor (SCF) [248]. The studies showed that VEGF plays an important role in neovascularization and angiogenesis during the development of most tissues including bone [249, 250].

Inflammation in damaged tissue stimulates macrophages and T lymphocytes for necrotic tissue phagocytosis and also induces inflammatory cytokines such as IL-1, IL-6, TNF-α, IFN-γ, monocyte chemoattractant proteins-1 (MCP-1), macrophage inflammatory protein-1 (MIP-1), and IL-17 [251, 252]. Although the inflammatory responses contribute substantially to bone regeneration, prolonged inflammation is harmful and retards the bone healing process [181]. BM, adipose tissue, CB, and WJ-derived MSCs cause reduction in IFN-γ and/or TNF-α secretion from T cells and suppress T cell proliferation [253]. In addition, MSCs significantly suppress the production of the inflammatory cytokines IL-6, IL-12p70, and IFN-γ while increase the production of anti-inflammatory cytokines IL-10 and IL-12p40 [254]. In response to inflammation and high levels of pro-inflammatory factors such as IFN-γ, TNF-α, and IL-1β, MSCs are stimulated to start producing anti-inflammatory factors such as nitric oxide (NO), indoleamine 2,3-dioxygenase (IDO), and anti-inflammatory cytokines and chemokines, which is followed by immunosuppression [255]. Three days after bone fracture, transplanted MSCs are capable of limiting tissue injury by significant reduction in IL-6, TNF-α, and IL-1β levels and preventing the progression of fibrosis and thus improve bone regeneration [256].

Another immunomodulatory mechanism of MSCs is inducing the polarization of monocytes into anti-inflammatory M2 macrophages through signal transducer and activator of transcription 3 (STAT-3), and NF-κB leading to indirectly suppression of T cell proliferation [257–259].

Toll-like receptors (TLRs) are highly expressed on MSCs and have profound effects on proliferation, migration, immunomodulatory functions, and survival of MSCs [229, 231, 260, 261]. TLR4 polarizes MSCs toward a pro-inflammatory phenotype (MSC1) which has critical role in early injury responses and lead to collagen deposition, expression of pro-inflammatory mediators, and reversal of the T cell suppressive mechanism [262]. In contrast, TLR3 supports the immune suppression and anti-inflammatory phenotype in MSCs, named MSC2 [263], which can suppress the T lymphocyte proliferation and induce regulatory T cell (Treg) generation by secreting soluble factors such as IDO, TGF-β1, and IL-6 [264]. In hAD-MSCs, activation of TLR2, TLR3, TLR4, and TLR9 leads to manganese superoxide dismutase (MnSOD) expression with an eminent impact on engraftment and survival of AD-MSCs in inflammatory conditions or injured tissues [265].

The most common therapeutic effects of MSCs are their incorporation into the host tissue and osteogenesis differentiation ability which are influenced by numerous cytokines and growth factors such as TGFβ-1 and WNT [266, 267]. Also, matrix metalloproteinases (MMPs) have critical role in the differentiation of MSCs to adipocytes, osteocytes, and chondrocytes. The MMP-1, MMP-13 (collagenase), and MMP-3 (Stromelysin-1) cleave ECM proteins [268]. The lowest production of MMP-1 and MMP-3 and no secretion of MMP-13 by BM-MSCs make them as suitable candidates for bone, cartilage, and tendon regeneration [269].

TNF-α, as a pro-inflammatory cytokine, is highly expressed in inflammatory sites of bone and causes tumor necrosis factor receptor1 (TNFR1) activation [270, 271] and receptor activator of nuclear factor kappa-Β ligand (RANKL) upregulation in osteoblasts [271]. Subsequently, NF-κB pathway is activated by receptor activator of nuclear factor kappa-Β (RANK)/RANKL signaling [272] which activates apoptotic factors including p21 and p53 [273, 274] and, as a result, inhibits MSC differentiation and increases apoptosis of osteoblasts and their progenitors [273]. Another important effect of TNF-α in inflammatory conditions is inhibition of two essential osteogenic differentiation factors including RUNX2 and osterix, leading to the suppression of MSC differentiation [275, 276]. It has been indicated that commitment of MSCs into the osteoblast lineage is regulated by Wnt/β-catenin signaling pathway [277]. β-catenin serves a notable role in the progression of MSC precursors differentiation into mature osteoblasts by upregulating the osteogenic regulators Runx2, Dlx5, and Osterix [278–280]. During inflammation, TNF-α suppresses Wnt/β-catenin signaling by inducing Wnt-signaling inhibitor, Dickkopf 1 (DKK1), and finally inhibits bone formation [281]. TNF-α which is released by activated immune cells such as T cells interacts with the TNF receptors on MSCs and leads to the production of prostaglandin E2 (PGE2), which then is the underlying reason of the suppression of T lymphocyte proliferation and consequently prevention of TNF-α expression [282, 283]. Also, IL-1RA released by MSCs induces IL-10 in stimulated DCs and inhibits TNF-α production by activated macrophages which results in accelerating bone healing [245].

Also, MSCs can express BMP-2 in defect site, which induces the differentiation of these cells into osteoblasts in an autocrine manner [177, 284]. BMP-2 plays an important role in bone healing due to the involvement in new bone tissue formation, increasing osteoblast function and the maintenance of the dynamic balance of the newly formed bone tissue [285, 286]. Through interacting with expressed BMP receptors, BMPs trigger two signal pathways including Smad-dependent pathways and the mitogen-activated protein kinase (MAPK) pathway, thereby involving in osteogenesis [287, 288].

The extracellular vesicles (EVs) produced by MSCs have been indicated as a novel therapeutic method for bone diseases such as osteoporosis. Exosomes are one of the most important EVs released by MSCs that can be directly used as therapeutic agents for various bone diseases [289]. The investigations indicated that exosomes secreted by MSCs promote osteoblast proliferation, differentiation, and bone formation, which improve bone regeneration in osteoporotic rats [290]. Through increasing the osteogenesis and angiogenesis-related genes expression, such as COL I, alkaline phosphatase (ALP), and VEGF, MSC-derived exosomes can promote bone formation [291]. In addition, exosomes contribute to bone repair and accelerate fracture healing through their cytokine content such as MCP-1, MCP-3, and SDF-1 [292].

**Fig. 3:**
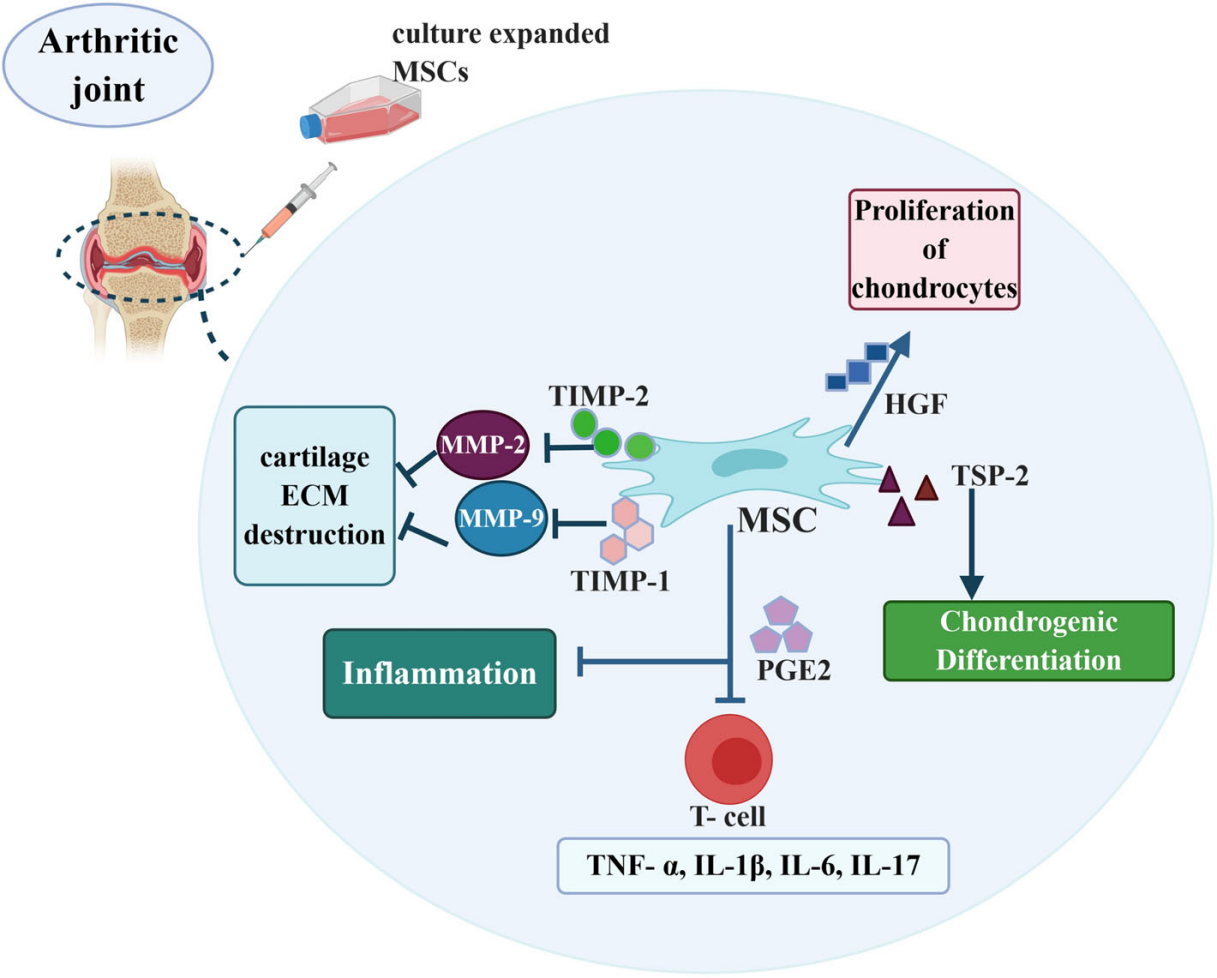
Mechanisms of MSC-mediated cartilage repair. The figure was designed using the web-based tool BioRender. Mesenchymal stem cells (MSCs) contribute to cartilage regeneration by several mechanisms including response to inflammation condition and differentiation through production of a variety of mediators. Matrix metalloproteinase (MMP), tissue inhibitors of metalloproteinases (TIMP), tumor necrosis factor alpha (TNF-α), prostaglandin E2 (PGE2), interleukin (IL), hepatocyte growth factor (HGF), thrombospondin (TSP2)

Numerous studies have demonstrated the successful MSC transplantation for healing of chondral lesions and repairing the damaged cartilage (Fig. 3). There are two main concepts for MSCs contribution to cartilage disease improvement: first, preventing the degradation of cartilage through the secretion of bioactive factors, and second, differentiating potential of MSCs to become chondrocytes [293, 294]. Pro-inflammatory cytokines including TNF-α, IL-6, IL-1β, and IL-17 play important roles in the development of pathological conditions in cartilage diseases [295]. MSCs modulate host immune responses by inhibiting the proliferation of T lymphocytes and pro-inflammatory cytokine secretion by prostaglandin E2 (PGE2) [296, 297].

MMP-2, MMP-9, and MMP-13 were detected at higher levels in human OA cartilage [298]. MSCs secrete high levels of tissue inhibitor of metalloproteinases 2 (TIMP2) and TIMP-1 inhibitors, which inhibit MMP-2 and MMP-9, respectively, and suppress cartilage ECM destruction [298]. MSCs secret hepatocyte growth factor (HGF) through which inhibit the fibrosis and apoptosis of chondrocytes, but stimulate the proliferation of these cells, and increase ECM synthesis [299]. The EVs produced by MSCs can reduce arthritis scores and pathological changes in inflamed cartilage by decreasing plasma blast population and increasing IL-10 secretion of regulatory B cells [300]. MSC’s exosomes can cause an early suppression of local inflammation in OA through a significantly reduced expression of inflammatory genes especially IL-1β [301, 302]. In vitro studies demonstrated that exosomes derived from BM-MSCs are able to stimulate the expression of chondrocyte markers such as type II collagen and aggrecan while inhibit MMP-13 and ADAMTS5 as catabolic markers in OA-like chondrocytes [303].

Different growth factors, cytokines, and signaling molecules including TGF-β superfamily regulate chondrogenic induction and differentiation of MSCs. TGF-β2, TGF-β1, and TGF-β3 stimulate the synthesis of collagen type II and proteoglycans and contribute to the MSC differentiation to chondrocytes [304, 305]. TGF-β signaling mediates chondrogenesis by activating and phosphorylating Smad2/3. Phosphorylated Smad translocates into the nucleus and binds to the master chondrogenic transcription factors such as SOX9 and collagen type II (COL II) which are expressed in all chondrocyte progenitors and chondrocytes [306, 307]. Other factors that influence MSC differentiation and chondrogenesis are Wnt/β-catenin signaling pathway family and MAP kinases [308, 309]. Thrombospondin (TSP2), as a regulator of cartilage and bone differentiation, is secreted by MSCs and promotes chondrogenic differentiation of progenitor cells by protein kinase C alpha (PKCα), extracellular signal-regulated kinase (ERK), p38/MAPK, and Notch signaling pathways [310, 311]. Moreover, some trophic factors such as VEGF, epidermal growth factor (EGF), and an array of bioactive molecules also affect chondrogenic differentiation from MSCs and cartilage matrix formation [312].

#### Selection of appropriate source of stem cell based on their protein expression profile

MSC therapy has been used for repairing both the structure and function of injured bone and cartilage tissues [158, 313]. In addition to differentiation capacity to the different cell types, MSCs obtained from various sources have diverse capabilities of secreting many different cytokines, growth factors, and chemokines and thus differentially influence angiogenesis, inflammation, apoptosis, stem cell homing, stem cell survival, proliferation potential, and migration to the damaged areas [314–317].

Amable et al. showed that WJ-MSCs have a higher proliferation potential, higher production of pro-inflammatory cytokines such as IL-6, and higher expression of some growth factors such as PDGF, HGF, and TGF-β compared with AD-MSCs and BM-MSCs. WJ-MSCs also produce a higher concentration of some pro-angiogenic proteins such as VEGF, ECM components such as collagen, and MMPs such as MMP1 and MMP3 in AD-MSC supernatants. In contrast, BM-MSCs secrete the lowest amount of all chemokines in comparison with stem cells from other sources [269]. Comparison of cytokine expression profile including macrophage migration inhibitory factor (MIF), IL-8, Serpin E1, growth-regulated oncogene α (GROα) and IL-6 in MSCs from human PL (amnion, decidua), cord blood (CB), and BM by Hwang et al. represented similar expression pattern in all three cell types. However, BM-MSCs express higher MCP-1 and are the only MSC type that produces SDF-1, but the expression of IL-6 by the CB-MSCs was comparatively lower [318].

hAD-MSCs, BM-MSCs, and UCB-MSCs express high levels of TLRs [319–321] compared with WJ-MSCs [228, 322]. One of the studies reported that human olfactory ecto MSCs (OE-MSCs) express high levels of TLR3 and TLR4 genes, as well as higher levels of cytokines and chemokines including CCL5, IL-8, and TGF-β in comparison with AD-MSCs [323]. In another study, perivascular stem cells derived from umbilical arteries (UCA-PSCs) and PSCs derived from umbilical vein (UCV-PSCs) showed higher expression of angiogenesis-related genes, such as CXCL12(SDF-1), HIF-1α, and ERAP1 in comparison with WJ-MSCs. In addition, higher expression of angiogenesis related genes such as CD146 and Jagged1 was detected in UCA-PSCs. Consequently, UCA-PSCs and UCV-PSCs, especially UCA-PSCs, demonstrated better angiogenic capability than WJ-MSCs [260].

Based on these investigations, identification of MSCs in terms of proteins expression and secretory factors has been of great benefit to appropriate cell source selection for each disease.

## Conclusion

Osteochondral complications promise as significant cause of disability and pain. Although the degenerative conditions are progressive, there has been no definitive therapy and almost all currently therapies try to control the symptoms. MSC-based therapy is introduced as a promising treatment strategy with potential ameliorating effects on disease progression. Despite using various sources of MSCs for bone defect therapy, BM-MSCs and AD-MSCs are widely applicable in human trials. Comparison of main sources of cellular tissue revealed that BM remains the most widely used source for bone fracture repair strategies as 14 of the 17 registered clinical trials have used BM-MSCs. However, both adipose tissue and bone marrow seem to be promising stem cell sources for osteoarthritis therapy. In addition, WJ-MSCs possess similar ECM components with cartilage and express cell growth factors, chemokines, and cytokines at levels similar to those of cartilage. Thus, they are appropriate cell candidates for osteoarthritis cell therapy. HUCB-MSCs are less immunogenic and have the chondrogenic differentiation potential, therefore promoting cartilage repair without bone formation in a long period of time. All in all, clinical trials have confirmed a relative safety of using MSCs in the treatment of osteochondral defects with both reparative and preventative effects rather than generally accepted pain managements. However, culturing and expanding these cells should be carried out with further caution and in controlled ex vivo preparation conditions.

## Data Availability

Not applicable.

## Abbreviations

MSCs: Mesenchymal stem cells
BM: Bone marrow
UC: Umbilical cord
UCB: Umbilical cord blood
PL: Placenta
WJ: Wharton’s jelly
IVDs: Intervertebral discs
OI: Osteogenesis imperfecta
OA: Osteoarthritis
RA: Rheumatoid arthritis
HA: Hyaluronic acid
GAGs: Glycosaminoglycans
DP: Dental pulp
hMSCs: Human MSCs
VEGF: Vascular endothelial growth factor
TGF-β: Transforming growth factor beta
ILs: Interleukins
DC: Dendritic cell
ECM: Extracellular matrix
BM-MSCs: Bone marrow-derived mesenchymal stem cells
NP: Nucleus pulposus
AF: Anulus fibrosus
CEPs: Cartilaginous end plates
MMPs: Matrix metalloproteinases
DDD: Degenerative disc disease
TNF-α: Tumor necrosis factor alpha
AD-MSCs: Adipose-derived mesenchymal stem cells
MdSCs: Muscle-derived stem cells
BMD: Bone mineral density
HF: Human fetal
IA: Intra-articular
IP: Intraperitoneal
IV: Intravenous
e-CSCs: Human fetal early chorionic stem cells
OPG: Osteoprotegerins
OSX: Osterix
BMP: Bone morphogenetic protein
CPCs: Calcium phosphate cements
β-TCP: β-tri-calcium phosphate
PLA: Polylactic acid
PCL: Poly(ε-caprolactone)
IGF-1: Insulin-like growth factor 1
CIA: Collagen-induced arthritis
IFN-γ: Interferon gamma
Tfh: T follicular helper
G-MSCs: Gingival tissue-derived MSCs
Tregs: Regulatory T cells
PDGFs: Platelet-derived growth factors
PDGFRα: Platelet-derived growth factor receptor-alpha
HIF-1α: Hypoxia-inducible factor 1-α
SDF-1: Stromal cell-derived factor-1
CXCR4: CXC chemokine receptor 4
PD-MSCs: Periosteum-derived mesenchymal stem cells
NF-κB: Nuclear factor kappa-B
SCF: Stem cell factor
MIP-1: Macrophage inflammatory protein-1
NO: Nitric oxide
IDO: Indoleamine 2,3-dioxygenase
STAT-3: Signal transducer and activator of transcription 3
MnSOD: Manganese superoxide dismutase
TNFR1: Tumor necrosis factor receptor1
PGE2: Prostaglandin E2
IL-1RA: Interleukin-1 receptor antagonist
MAPK: Mitogen activated protein kinase
TIMP-2: Tissue inhibitor of metalloproteinases-2
EGF: Epidermal growth factor
HGF: Hepatocyte growth factor
MIF: Macrophage migration inhibitory factor
GROα: Growth-regulated oncogene α
CB: Cord blood
MCP-1: Monocyte chemoattractant protein-1
TLRs: Toll-like receptors
OE-MSCs: Olfactory ecto MSCs
UCA-PSCs: Perivascular stem cells derived from umbilical arteries
UCV-PSCs: Perivascular stem cells derived from umbilical vein
